# A methodological framework for assessing agreement between cost-effectiveness outcomes estimated using alternative sources of data on treatment costs and effects for trial-based economic evaluations

**DOI:** 10.1007/s10198-017-0868-8

**Published:** 2017-02-09

**Authors:** Felix Achana, Stavros Petrou, Kamran Khan, Amadou Gaye, Neena Modi, On behalf of Medicines for Neonates Investigators, Peter Brocklehurst, Jane Abbott, Kate Costeloe, Elizabeth Draper, Azeem Majeed, Jacquie Kemp, Deborah Ashby, Alys Young, Stavros Petrou

**Affiliations:** 1 Clinical Trials Unit, Warwick Medical School, University of Warwick, Coventry, CV4 7AL UK; 2 National Institutes of Health, National Human Genome Research Institute, Bethesda, MD 20892 USA; 3 Section of Neonatal Medicine, Department of Medicine, Chelsea and Westminister Hospital Campus, Imperial College, London, SW10 9NH UK

**Keywords:** Agreement, Cost-effectiveness analysis, Economic evaluation, Routine data, Electronic health records, I

## Abstract

A new methodological framework for assessing agreement between cost-effectiveness endpoints generated using alternative sources of data on treatment costs and effects for trial-based economic evaluations is proposed. The framework can be used to validate cost-effectiveness endpoints generated from routine data sources when comparable data is available directly from trial case report forms or from another source. We illustrate application of the framework using data from a recent trial-based economic evaluation of the probiotic* Bifidobacterium breve* strain BBG administered to babies less than 31 weeks of gestation. Cost-effectiveness endpoints are compared using two sources of information; trial case report forms and data extracted from the National Neonatal Research Database (NNRD), a clinical database created through collaborative efforts of UK neonatal services. Focusing on mean incremental net benefits at £30,000 per episode of sepsis averted, the study revealed no evidence of discrepancy between the data sources (two-sided *p* values >0.4), low probability estimates of miscoverage (ranging from 0.039 to 0.060) and concordance correlation coefficients greater than 0.86. We conclude that the NNRD could potentially serve as a reliable source of data for future trial-based economic evaluations of neonatal interventions. We also discuss the potential implications of increasing opportunity to utilize routinely available data for the conduct of trial-based economic evaluations.

## Introduction

In trial-based economic evaluations, data on treatment costs and consequences (effects) are required for trial participants with the aim of estimating the relative cost-effectiveness of two or more interventions. It is common practice within this context for multiple sources of information to be obtained by analysts and used to inform the evaluation. For example, data on healthcare resource use and costs can normally be obtained from a variety of sources including trial case report forms, medical records, patient questionnaires, and diaries [26]. With the advent of the ‘big data’ revolution, large volumes of individual-level information are being collected prospectively from patients and stored in administrative datasets and electronic health record systems. These routinely collected datasets constitute a rich source of information for health research—they are increasingly being relied upon as sources of information for trial-based economic evaluations and health technology assessments. For example, data drawn from the Hospital Episode Statistics (HES) in the UK have been obtained for use in the CAP trial to evaluate the clinical and cost-effectiveness of prostate-specific antigen testing in men aged 50–69 years old [36, 37]. Furthermore, in a recently published editorial in the *British Medical Journal* on reforms of the UK Cancer Drug Fund, Grieve et al. [13] suggested “using timely randomized controlled trials within routinely collected data sources, to establish which drugs are relatively effective” and cost-effective.

It is likely that use of routine data in trial-based economic evaluations will increase in the coming years in the UK context and beyond. This is largely driven by increased access to datasets and advances in computerized record linkage that enable datasets to be linked with each other [29] and increasingly to trial participants at the individual patient-level. Linkage to trial participants is crucial in this context as the within-trial randomization can be relied upon to generate unbiased estimates of treatment impacts based on information contained in the routine data sources. That being said, what is not known is whether routine data sources can provide reliable information across the broad array of data required for trial-based economic evaluations [36]. This is because the datasets have generally been compiled for non-research purposes, such as the need to evaluate health service performance or monitor care delivery, and hence may not adequately satisfy the rigors required of clinical trial research. Consequently, there are often concerns about data quality, including missing information, incomplete coding, and miss-classification of variables—issues that have potential to render the data unsuitable for most clinical research.

For the reasons stated above, analysts working on trial-based economic evaluations have long recognized the need for validated data obtained from disparate sources for application within their evaluations [4]. In this context, analysts have examined the disparate sources of information for evidence of difference [36] or agreement [4, 15, 21, 23, 31] in individual parameter estimates. These studies have primarily focused on comparisons between multiple sources of information on individual-level healthcare resource use or costs.

In this paper, we outline a new methodological framework for assessing agreement between the final cost-effectiveness endpoints generated using alternative sources of data on treatment costs and effects for trial-based economic evaluations. The proposed framework builds on the earlier work of Bland and Altman [1, 3] and Lin [18] on methods for assessing the reproducibility of clinical assays, measurements, and tests. The framework can be used to validate estimates of cost-effectiveness endpoints generated using routine data sources when comparable data on costs and effects for trial participants are available from a de novo data source, such as trial case report forms.

Of the two most commonly reported endpoints in economic evaluations, namely the incremental cost-effectiveness ratio (ICER) and the incremental net-benefit statistic, we base our assessment of agreement on the latter. This is because of well-known issues surrounding the ICER [12, 33] that makes it unsuitable as a statistic on which to base assessment of agreement. For example, the sampling distribution of the ICER is unknown, and it can be problematic to estimate associated measures of uncertainty. Also, because the ICER is a ratio of incremental costs and incremental effects, two ICERs can be equal in magnitude but qualitatively different in meaning when they fall in different quadrants of the cost-effectiveness plane. The incremental net benefit statistic, on the other hand, is unambiguous with relatively straightforward interpretation and its sampling distribution is known at the specified cost-effectiveness threshold [33].

The remainder of the paper is structured as follows: “Methods” outlines the proposed methodological framework. In “Example application to the PiPS trial”, we illustrate an application using data from a recently conducted trial-based economic evaluation investigating the benefits of early administration of the probiotic* Bifidobacterium breve* strain BBG (B breve BBG) to prevent development of infection (sepsis) in babies less than 31 weeks of gestation. We present final concluding remarks in the “Discussion” section, including the potential implications of increasing recourse to routinely collected data for the conduct of trial-based economic evaluations.

## Methods

This section outlines our framework for assessing agreement between the mean incremental net (monetary) benefits estimated from two sets of data on treatment costs and effects for trial participants. Three commonly used statistics are adapted for this purpose: (1) the mean difference; (2) the probability estimate of miscoverage; and (3) the concordance correlation coefficient [18] between two estimates of the incremental net benefit. We define the probability estimate of miscoverage as the proportion of samples in simulated replication of trial data in which the confidence limits for the mean incremental net benefit from one data source, designated as test data, contain the mean incremental net benefit estimated from the second data source, designated as the referent or gold standard data source. We outline a strategy for estimating the miscoverage probability and show how the concordance correlation coefficient can be adapted for assessing agreement between two estimates of the mean incremental net benefit evaluated at a specified cost-effectiveness threshold. A package to implement the routines described in the remainder of the paper in [28] is available from https://github.com/agaye/ceeComp.

### Difference between two estimates of the incremental net benefit

Consider a trial in which paired data on treatment costs and effects, denoted as *D*_*1*_ and *D*_*2*_, are available for *N* trial participants randomized to one of two interventions, denoted as *A* and *B*. Our illustrative example in the section “Example application to the PiPS trial” highlights two potential data sources, namely trial case report forms and data obtained from a national patient electronic system. Denote *A* as control intervention and let $\beta_{i\lambda }$ be an estimate of the mean incremental net benefit of intervention *B* relative to *A* from the *i*th dataset *D*_*i*_ $(i = 1,\,2)$ at a specified cost-effectiveness threshold $\lambda$. Then a simple measure of discrepancy between the two estimates of cost-effectiveness (in the form of the incremental net benefit of intervention *B* relative to *A*) generated from two data sources is $\omega_{\lambda }$ where

1 $$\omega_{\lambda } = \beta_{2\lambda } - \beta_{1\lambda } .$$

The variance of $\omega_{\lambda }$ (after dropping the $\lambda s$ to simplify the notation) is given by

2 $$\sigma_{\omega }^{2} = \sigma_{{\beta_{1se} }}^{2} + \sigma_{{\beta_{2se} }}^{2} - 2\rho_{{\beta_{1se} ,\beta_{2se} }} ,$$

where $\sigma_{{\beta_{1se} }}$, $\sigma_{{\beta_{2se} }}$ represent standard error of the incremental net benefit from datasets 1 and 2, respectively. Incremental net benefits generated this way are likely to be correlated, as the two datasets contain information from the same patients, the parameter $\rho_{{\beta_{1se} ,\beta_{2se} }}$ quantifies the covariance between the two. The parameters $\omega$, $\beta_{1}$, $\beta_{2}$ and associated variance and covariance terms in Eqs. (1) and (2) are unobserved, hence will be replaced in practice with their sample counterparts $\hat{\omega }$, $\hat{\beta }_{1}$ and $\hat{\beta }_{2}$, respectively. We show in Appendices A and B that the variance and covariance terms on the right hand side of Eq. (2) can be written in terms of the variance of costs and effects and the covariance between the two within the respective arms of a trial with parallel group design (assuming no treatment switching or cross-over effects common in cancer trials). Under the large sample assumption, an approximate statistical test of the null hypothesis that there is no difference between incremental net benefits generated from the two data sources (i.e., $\omega = 0$) can be constructed by referring an estimate $\hat{Z}$ of the *Z* statistic to the standard normal distribution where $\hat{Z}$ is given by

3 $$\hat{Z} = \frac{{\hat{\omega }}}{{\hat{\sigma }_{\omega } }}.$$

Note that failure to reject the null hypothesis of no agreement above does not imply evidence of agreement or that the two incremental net benefits are equivalent. A statistical test of equivalence if required can be constructed by specifying an equivalence margin *δ* followed by two one-sided tests of the hypothesis that* |ω| < ± δ *[38].

### Probability of miscoverage

This section introduces the probability estimate of miscoverage as a statistic for assessing agreement between two cost-effectiveness estimates. Miscoverage probabilities have previously been used in the health economics literature [27] to compare the performance of different methods for estimating confidence intervals for the ICER. However, unlike Polsky et al., we base our assessment on the incremental net benefit rather than the ICER for the reasons stated in the introduction. For any two data sources that are available for the economic evaluation, we first designate one data source as referent data and the other as test data. From the referent dataset, we calculate $\hat{\beta }_{ref,\lambda }$, the sample estimate of the underlying population mean incremental net benefit $\beta_{ref,\lambda }$ at cost-effectiveness threshold $\lambda$. Next, we sample with replacement several times to generate *S* bootstrap replicates of the test data. For each replicate dataset, we calculate a bootstrap estimate of the incremental net benefit and the associated variance given by Eq. (9) of Appendix A. Finally, we obtain the probability of miscoverage by counting the proportion of the *S* bootstrap replicates in which the (95%) confidence intervals for the incremental net benefit statistic does not contain the corresponding estimate from the referent dataset.

### Concordance correlation

Lin [18] introduced the concordance correlation coefficient, $\rho_{c}$ and used it to quantify agreement or reproducibility of a clinical assay, test, or measuring instrument compared to the current measure or a gold standard. In doing so, Lin [18–20] defined perfect agreement between two measurements as a 45° line passing through the origin of the Cartesian (*X*, *Y*) plane so that deviations from this line indicate evidence of disagreement. The concordance correlation coefficient quantifies this deviation in terms of the precision and accuracy of the new measure compared to the gold standard. As a correlation coefficient, $\rho_{\text{c}}$ satisfies the inequality $- 1 \le \rho_{\text{c}} \le 1$ where $\rho_{\text{c}} = 1$ indicates perfect agreement, $\rho_{\text{c}} = 0$ no agreement and $\rho_{\text{c}} = - 1$ perfect inverse agreement.

To adapt Lin’s method for our purpose, let $\left( {D_{j1} = \left\{ {C_{j1} ,E_{j1} ,t_{j} } \right\},D_{j2} = \left\{ {C_{j2} ,E_{j2} ,t_{j} } \right\}} \right)$ denote again our paired outcome information (comprising of treatment costs $C_{jt}$ and effects $E_{jt}$) for the *j*th patient ($j = 1,2, \ldots ,N$) in treatment group $t_{j} = A\,or\,{B}$ from a bivariate population with mean incremental net monetary benefit $(\beta_{1} ,\beta_{2} )$ and variance $(\sigma_{{\beta_{1} }}^{2} ,\sigma_{{\beta_{2} }}^{2} )$ at specified cost-effectiveness threshold. Following Lin [18], the degree of concordance between incremental net-benefits generated from the two data sources can be quantified by the expected value of the squared difference on the incremental net benefit scale:

4 $$E[(D_{2} - D_{1} )^{2} ] = (\beta_{2} - \beta_{1} )^{2} + \sigma_{{\beta_{1} }}^{2} + \sigma_{{\beta_{2} }}^{2} - 2\rho_{{\beta_{1} \beta_{2} }}.$$

where $\sigma_{\beta1}\, \text{and} \,\sigma_{\beta2}$ represent standard deviation of incremental net benefit generated from the two datasets and $\rho_{{\beta1}{\beta2}}$ are the covariance between the two. Lin [18] showed that Eq. (4) can be written in terms of the Pearson correlation coefficient $\rho$ which he suggested provided a measure of precision (i.e., “how far each observation deviates from the best fitted line”) and a bias correction factor $C_{\text{b}}$ that measures accuracy (i.e., “how far the best fitted line deviates from the 45° line”):

$$\rho_{\text{c}} = \rho C_{\text{b}} \quad {\text{where}}\quad C_{\text{b}} = \frac{{2\sigma_{{\beta_{1} }} \sigma_{{\beta_{2} }} }}{{(\beta_{2} - \beta_{1} )^{2} + \sigma_{{\beta_{1} }}^{2} + \sigma_{{\beta_{2} }}^{2} }}.$$

when used to assess agreement between pairs of measurements, an estimate $\hat{\rho }_{\text{c}}$ of $\rho_{\text{c}}$ is obtained by replacing the parameters in Eq. (4) with their sample estimates. Hence, in our adaptation of Lin’s method, we define $\hat{\rho }_{\text{c}}$ in terms of the incremental net benefit generated from two data sources:

5 $$\hat{\rho }_{\text{c}} = \frac{{2\hat{\rho }_{{\beta_{1} ,\beta_{2} }} }}{{(\hat{\beta }_{2} - \hat{\beta }_{1} )^{2} + \hat{\sigma }_{{\beta_{1} }}^{2} + \hat{\sigma }_{{\beta_{2} }}^{2} }},$$

where $\hat{\beta }_{2}$ and $\hat{\beta }_{1}$ represent sample estimates of the incremental net benefit from the respective datasets, $\hat{\sigma }_{{\beta_{1} }}$ and $\hat{\sigma }_{{\beta_{2} }}$ represent estimates of the corresponding standard deviation and $\hat{\rho }_{{\beta_{1} ,\beta_{2} }}$ estimate of the covariance between the two. Again as shown in Appendices A and B, the parameters on the right-hand side of Eq. (5) can be written in terms of the arm-specific estimates of the mean costs and effects given by Eq. (6) and associated variance and covariance terms given by Eqs. (9) and (16), respectively. Finally, to estimate a confidence interval and carry out hypotheses tests, Lin [18] suggested the Fisher *Z* transformation as a useful approximation to the standard normal distribution with mean

$$Z_{{\rho_{\text{c}} }} = \frac{1}{2}\ln \left( {\frac{{1 + \rho_{\text{c}} }}{{1 - \rho_{\text{c}} }}} \right),$$

and variance $\sigma_{{Z_{{\rho_{\text{c}} }} }}^{2}.$ An estimate $Z_{{\hat{\rho }_{\text{c}} }}$ and $\sigma_{{Z_{{\hat{\rho }_{\text{c}} }} }}^{2}$ of $Z_{{\rho_{\text{c}} }}$ and $\sigma_{{Z_{{\rho_{\text{c}} }} }}^{2}$ can be obtained using bootstrapping before re-transforming back to the original scale.

Statistical tests of the hypothesis that $\rho_{\text{c}}$ is greater than an arbitrarily defined threshold value, $\rho_{{{\text{c}}0}}$, can be constructed using the transformed parameters and one-sided* p* values generated for a specified level of significance. Concordance correlation coefficient thresholds often cited in the literature as indicating acceptable levels of agreement include $\rho_{\text{c0}} > 0.4$ [4] and $\rho_{{{\text{c}}0}} > 0.65$ [11] with coefficients greater than 0.8 generally taken as good evidence of agreement [11, 22]. Rather than define an arbitrary threshold value, an alternative strategy suggested by Lin [19] is to estimate $\rho_{{{\text{c}}0}}$ through the expression $\rho_{\text{c0}} = C_{\text{b}} \sqrt {\rho^{2} - x}$ where $x$ represents a pre-specified percentage loss in precision that is acceptable for the particular measure or clinical scenario under investigation and $\rho$ is again the Pearson correlation coefficient. For example, $x = 0.05$ for a 5% acceptable loss in precision. In our adaptation of this approach, if we designate one dataset as the referent data and another as the test dataset, then $x$ represents the percentage loss in precision in mean incremental net monetary benefit generated from the test data that can be considered acceptable compared with the corresponding estimate obtained using the referent dataset. Statistical tests of the hypothesis that $\rho_{\text{c}} > \rho_{\text{c0}}$ can then be constructed and one-sided* p*-values estimated.

## Example application to the PiPS trial

### Example data

The Probiotic in Preterm babies Study (PiPS) is a multi-center, double-blind, placebo-controlled randomized trial of probiotic administration in infants born between 23^+0^ and 30^+6^ weeks gestational age. The trial recruited 1300 infants within 48 h of birth from 24 hospitals within 60 miles of London over a 37-month period from July 2010 onwards. Infants were randomized to receive either the probiotic *Bifidobacterium breve* BBG-001 or a matching placebo. Details of the trial design and baseline characteristics of trial participants are published elsewhere [7]. The main trial analyses and findings have also been published [6]. The main trial economic evaluation has not yet been published, so a summary of the methods used to conduct the evaluation is presented in Appendix C. For the purpose of illustrating the methodology described in this paper, we restrict ourselves to 1258 of the 1300 infants who had complete data on treatment costs and clinical outcomes of interest. Of these, 638 infants were in the placebo group and 620 in the probiotic group. Three clinical outcomes were considered in the trial: (1) any episode of neonatal necrotizing enterocolitis (NEC) Bell stage 2 or 3 [2]; (2) any positive blood culture of an organism not recognized as a skin commensal on a sample drawn more than 72 h after birth and before 46 weeks postmenstrual age or discharge if sooner (hereafter referred to as sepsis for brevity); and (3) death before discharge from hospital. We restrict ourselves to the sepsis outcome for the purpose of illustrating the methodological framework described in this paper.

Data on PiPS trial participants were available from two primary sources, the trial case report forms and the National Neonatal Research Database (NNRD) (The Neonatal Data Analysis Unit [34]. The PiPS trial case report forms captured a comprehensive profile of resource use by each infant, encompassing length of stay by intensity of care, surgeries, investigations, procedures, transfers and post-mortem examinations until final hospital discharge or death (whichever was earliest). Resource inputs were primarily valued based on data collated from secondary national tariff sets [8]. All costs were expressed in pounds sterling and reflected values for the financial year 2012–13. The trial case report forms also captured information on the clinical outcomes of interest. The NNRD has been created through the collaborative efforts of neonatal services across the UK to be a national resource. The NNRD contains a defined set of data items (the Neonatal Dataset) that have been extracted from the Badger.net neonatal electronic patient record of all admissions to National Health Service (NHS) neonatal units. Badger.net is managed by Clevermed Ltd, an authorized NHS hosting company. The Neonatal Dataset is an approved NHS Information Standard (ISB1575) and contributing neonatal units are known as the UK Neonatal Collaborative.

Our comparisons of cost-effectiveness outcomes were based on four datasets that we created using information from the two primary data sources: (1) the trial case report forms as the sole source of information (hence forth referred to as PiPS dataset); (2) the NNRD as the source of information on resource inputs only with clinical outcomes extracted from the PiPS case report forms (herein referred to as the NNRD1 dataset); (3) the NNRD as a source of resource use and clinical outcomes (herein referred to as the NNRD2 dataset); and (4) a combined dataset created by the selection of a preferred data source (by clinical experts) for each data input.

### Results

**Fig. 1:**
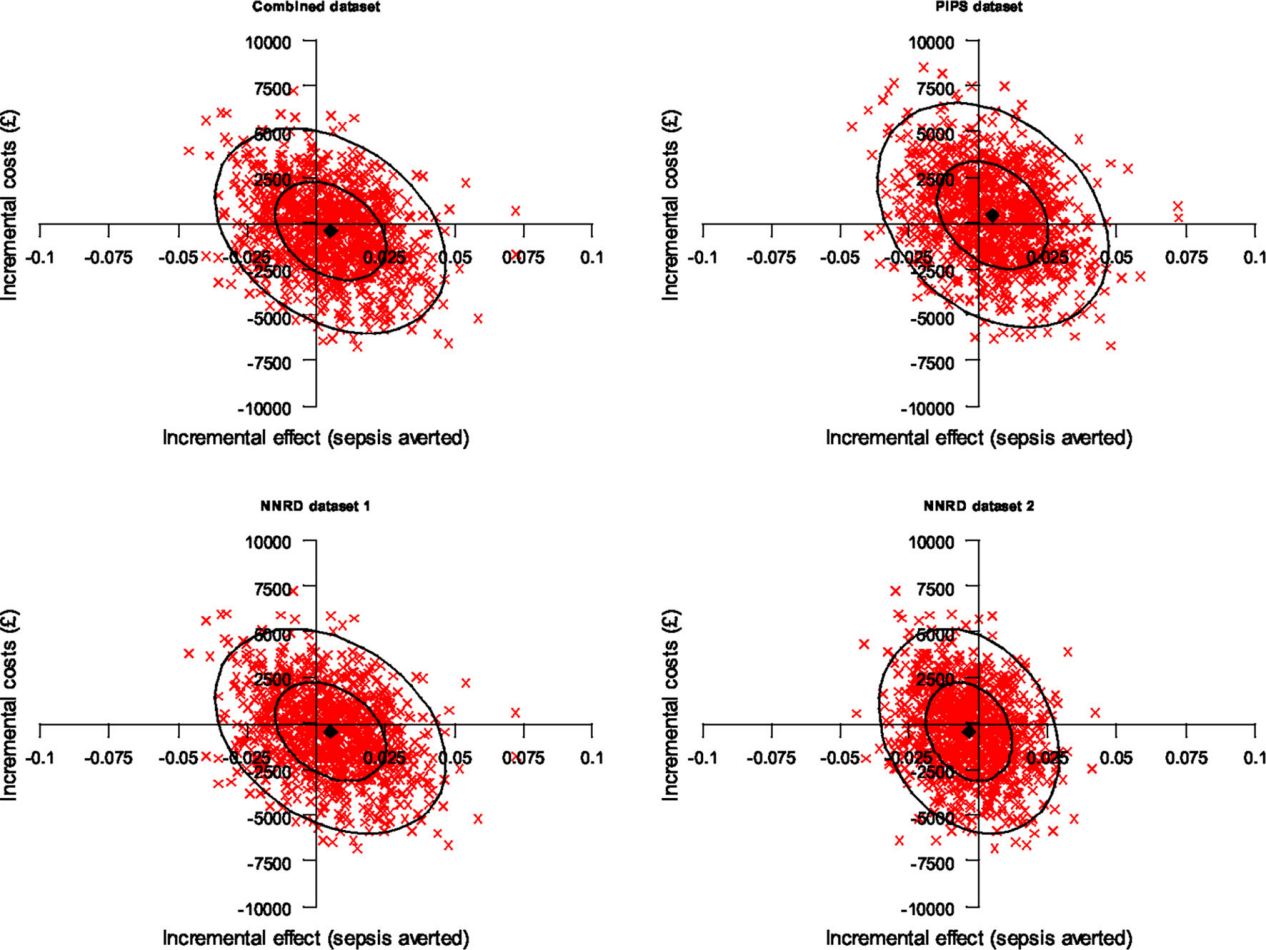
PIPS trial ICERs from the four datasets comparing probiotic versus placebo for prevention of sepsis in newborn infants displayed on the cost-effectiveness plane. *NNRD1 dataset* acted as source of resource use information only. *NNRD2* acted as source of both resource use and clinical outcome information

**Fig. 2:**
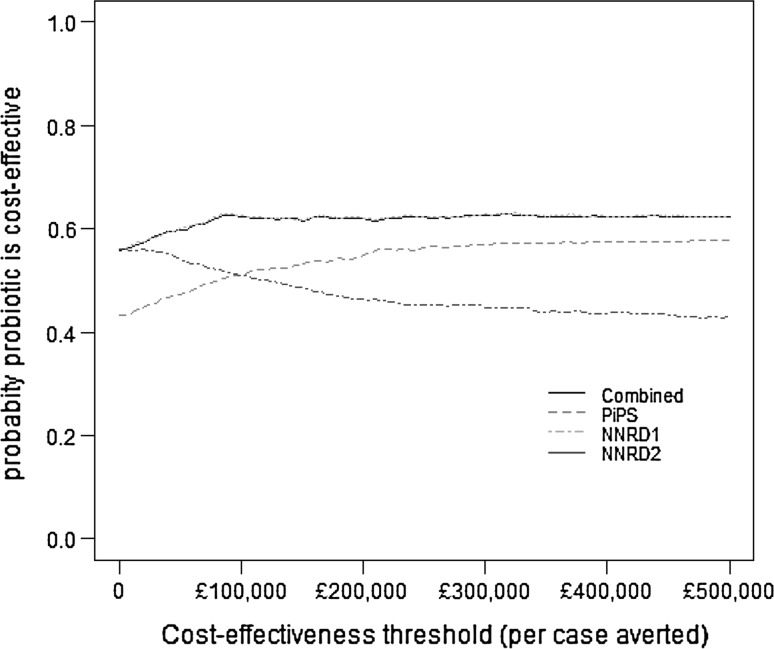
Cost-effectiveness acceptability curves indicating probability at which the probiotic is cost-effective compared with placebo for a range of cost-effectiveness or willingness-to-pay thresholds

Table 1 presents descriptive summaries of the cost-effectiveness estimates for the probiotic compared to placebo, obtained from each of the four datasets described above. Based on the data from the trial case report forms (PiPS dataset), the proportion of infants with sepsis and the mean total cost were 10.8% and £62,799, respectively, in the probiotic group, compared with 11.3% and £62,284 in the placebo group, generating a mean absolute incremental effect of 0.50%, mean incremental costs of £515, and an ICER of £107,613 per episode of sepsis averted. As stated above, the trial case report forms also served as the primary source of clinical outcome information for the NNRD1 and the combined datasets, thus these two datasets differed from the PiPS dataset only in terms of healthcare utilization data and hence treatment costs. For these (NNRD1 and the combined) datasets, the probiotic was associated with slightly lower total healthcare costs than placebo, generating a mean cost saving of £367 in the NNRD1 dataset and £342 in the combined datasets. Thus, on average, the probiotic dominated placebo in health economic terms in these two datasets. Finally, the NNRD2 dataset indicated that the probiotic is less effective and less costly, on average, than placebo, generating a mean ICER of £111,348 per episode of sepsis averted for the probiotic compared with placebo. Overall, although the PiPS and NNRD2 datasets generated mean ICERs that are very similar in magnitude, they have different interpretations because the mean ICER for the PiPS dataset occupies the north-east quadrant of the cost-effectiveness plane, suggesting that the probiotic is more costly and more effective than placebo, whereas the mean ICER for the NND2 dataset occupies the south-west quadrant where the probiotic is less costly but also less effective (Fig. 1). The mean ICERs from three of the four datasets fell in a different quadrant of the cost-effectiveness plane, but a large proportion of the simulated ICERs from each dataset fell in all four quadrants, reflecting the considerable uncertainty surrounding the mean ICERs. Figure 1 illustrates the point made in the introduction that the ICER may not be an appropriate statistic for assessing agreement between estimates of cost-effectiveness generated from alternative data sources. Cost-effectiveness acceptability curves based on three of the four datasets indicates the probiotic is the most cost-effective strategy for sepsis prevention in pre-term infants with probability of 0.6 but only at considerably high cost-effectiveness thresholds (upwards of £80,000 per sepsis avoided) whilst the probiotic is dominated by placebo in the NNRD2 dataset (Fig. 2). Overall, the results suggest the probiotic is not cost-effective unless policy makers are willing to spend large amounts of money to prevent infants from developing sepsis.

**Table 1:** Cost-effectiveness results from the PiPS trial datasets

Dataset^a^	Placebo arm		Probiotic arm		Cost-effectiveness		
	Costs (£)	Outcome^b^	Costs (£)	Outcome^b^	Incremental costs (95% confidence interval)^c^	Incremental effects (95% confidence interval)^d^	ICER
PiPS	62,284 (1876)	0.113 (0.013)	62,799 (1817)	0.108 (0.013)	515 (−4603, 5633)	0.005 (−0.03, 0.039)	107,613
NNRD1	60,927 (1805)	0.113 (0.013)	60,560 (1571)	0.108 (0.013)	−367 (−5058, 4323)	0.005 (−0.03, 0.039)	−76,662
NNRD2	60,927 (1805)	0.058 (0.009)	60,560 (1571)	0.061 (0.010)	−367 (−5058, 4323)	−0.003 (−0.029, 0.023)	111,348
Combined	60,796 (1799)	0.113 (0.013)	60,454 (1566)	0.108 (0.013)	−342 (−5016, 4332)	0.005 (−0.03, 0.039)	−71,422

*PiPS dataset* trial case report forms as the sole source of information, *NNRD1 dataset* NNRD as the source of information on resource inputs only with clinical outcomes extracted from the PiPS case report forms, *NNRD2 dataset* NNRD as a source of resource use and clinical outcomes, *Combined dataset* combined dataset created by the selection of a preferred data source (by clinical experts) for each data input

^a^Datasets

^b^Outcome = proportion of sepsis

^c^Incremental costs (£) is defined as mean costs in probiotic arm minus mean costs in placebo arm

^d^Incremental effects is proportion of sepsis avoided, hence effectiveness differential is reversed (i.e., mean effect in placebo arm minus mean effect in the probiotic arm) because the outcome is an adverse event

Table 2 presents the agreement statistics (mean difference, probability estimates of miscoverage and concordance correlation coefficients) between estimates of the mean incremental net benefit from combinations of the four alternative datasets using a cost-effectiveness threshold of £30,000 per episode of sepsis avoided. At this threshold, the probability estimate of miscoverage was very small, ranging from 3.9% when the combined dataset acted as referent source and the NNRD1 acted as the test data to 6.0% when the PiPS dataset acted as referent and the NNRD2 as the test data. The corresponding* p* values ranged from 0.387 for the comparison between the PiPS versus NNRD1 datasets to 0.634 for the comparison between the PiPS versus NNRD2 datasets. These results thus provide no evidence to suggest that the incremental net benefit estimated using one dataset is significantly different from the incremental net benefit estimated from the other datasets at a cost-effectiveness threshold of £30,000 per episode of sepsis avoided.

**Table 2:** Statistics comparing the agreement between cost-effectiveness estimates from the PiPS trial datasets

Comparison quadrant		Agreement statistics							
		Difference in means				Probability of miscoverage^‡^	Concordance correlation		
Dataset 1	Dataset 2	Mean INB (std. err) from dataset 1	Mean INB (std. err) from dataset 2	MD (SE)	*p* value^†^		$\rho_{c}$ (95% CI)	$\rho_{c0}$ ^a^	*p* value^††^
PiPS	NNRD1	−372 (2808)	511 (2596)	882 (1021)	0.387	0.060	0.882 (0.870, 0.893)	0.856	<0.001
PiPS	NNRD2	−372 (2808)	268 (2520)	640 (1129)	0.571	0.051	0.885 (0.874, 0.895)	0.858	<0.001
NNRD^1^	NNRD2	511 (2596)	268 (2520)	−243 (454)	0.593	0.041	0.980 (0.977, 0.982)	0.954	<0.001
Combined	PiPS	486 (2588)	−372 (2808)	−857 (1021)	0.401	0.049	0.884 (0.872, 0.895)	0.858	<0.001
Combined	NNRD1	486 (2588)	511 (2596)	25 (44)	0.565	0.046	1.000 (1.000, 1.000)	0.974	<0.001
Combined	NNRD2	486 (2588)	268 (2520)	−217 (457)	0.634	0.039	0.980 (0.978, 0.983)	0.955	<0.001

*INB* incremental net benefit evaluated at willingness-to-pay threshold of £30,000 per adverse event averted, *Std. err.* Standard error of the estimate, *MD* difference between mean *INB* from dataset 1 and mean *INB* from dataset 2, $\rho_{c}$ *(95% CI)* concordance correlation coefficient (95% confidence intervals) between the incremental net benefits at threshold of £30,000 per adverse event averted

^†^Two-sided *p* value at 5% significance level

^‡^The first dataset in each pairwise comparison is designated as referent when estimating the probability of miscoverage

*p* value^††^ One-sided test of the hypothesis that $\rho_{c} > \rho_{c0}$ where $\rho_{c0}$ is the least acceptable concordance correlation coefficient assuming 5% ($\rho_{c0}$ ^a^). A *p* value greater than 0.025 suggests significant evidence of disagreement at the at the 5% significance level

Agreement between mean incremental net benefit statistics from alternative datasets as measured by the concordance correlation coefficient ranged from a correlation coefficient of 0.882 (95% CI 0.870–0.893) for the comparison between the PiPS and the NNRD1 datasets to a coefficient of 1 indicating perfect correlation for the comparison between the combined and the NNRD1 datasets at the £30,000 per episode of sepsis avoided threshold. These correlation coefficients are well above the commonly cited threshold of 0.4 commonly taken as indicating evidence of good agreement [4]. The alternative strategy is to define a threshold based on percentage loss in precision that is acceptable for the clinical issue being investigated. Estimates of $\rho_{{{\text{c}}0}}$ based on a 5% loss in precision criterion ranged from 0.856 for the PiPS versus NNRD2 comparison to 0.975 for the combined versus NNRD1 comparison. These values of $\rho_{{{\text{c}}0}}$ were significantly lower than the lower confidence limit for $\rho_{\text{c}}$ (*p* < 0.0001) in each pairwise comparison (Table 2), indicating stronger evidence of agreement between datasets.

**Fig. 3:**
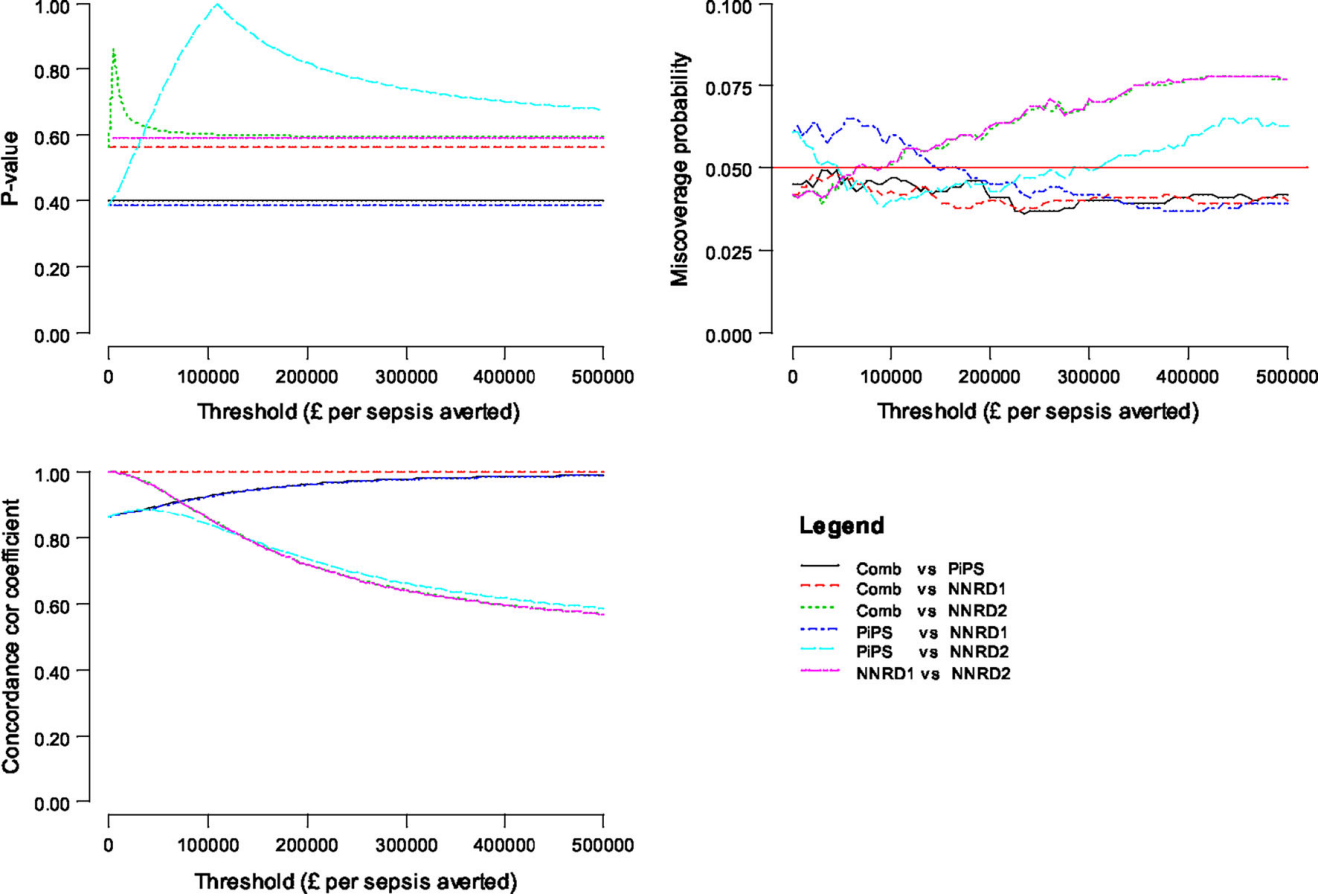
Two-sided *p* values, probability estimates of miscoverage and concordance correlation coefficients for comparing the agreement between cost-effectiveness estimates from the PiPS, NNRD, and combined data sources. *NNRD1 dataset* acted as source of resource use information only*. NNRD2* acted as source of both resource use and clinical outcome information

Estimates of the agreement statistics at cost-effectiveness thresholds between £0 and £500,000 per episode of sepsis avoided were also generated and can be read off the plots in Fig. 3. The *p* values remained relatively constant across different values of $\lambda$ for pairwise comparisons between the PiPS, NNRD1, NNRD2, and the combined datasets. Although no attempt was made to correct for multiple testing at different thresholds, this can easily be achieved by for example, defining a statistical significance at the 1% level instead of the 5% level [36]. Overall, across cost-effectiveness thresholds ranging from £0 to £500,000 per sepsis avoided and for all pairwise comparisons between datasets, differences between mean incremental net benefits were not statistically significant (*p* values ≥0.4), the probability estimates of miscoverage fell within the interval (0.025–0.075) and concordance correlation coefficient were greater than 0.5.

## Discussion

In this paper, we have shown how three commonly used metrics (namely difference in mean, miscoverage probability, and the concordance correlation coefficient) can be adapted and used to assess agreement between the final economic endpoints generated from alternative sources of data on costs and effects within the context of trial-based economic evaluations. Agreement statistics are obtained for a range of cost-effectiveness thresholds and plotted on simple graphs to ease comparability. Application of the method to data from the PiPS trial datasets revealed no evidence of disagreement, low probability levels of miscoverage, and high concordance correlation between estimates of incremental net monetary benefit generated using data from trial case report forms and data from the NNRD dataset.

Assessment of agreement in the health economics literature [4, 21, 23] have thus far focused on comparisons between alternative sources of resource use and cost variables, primarily because healthcare utilization data can and has often been collected from a multitude of sources such as patient self-reports, medical records, and trial case report forms. Data on clinical endpoints have, however, tended to come from a single source, often the trial case report forms. With recent advances in data management and information sciences, routine datasets are increasingly being compiled that have potential to provide patient-level resource utilization and clinical outcomes data for trial-based economic evaluations. As these potentially rich sources of data become available for clinical research, methods for assessing the level of agreement between final cost-effectiveness outcomes (of interest in the trial-based economic evaluations) generated using alternate sources of data will be of interest to analysts working on health economic evaluations and health technology assessments. We have shown how such assessments can be carried out in practice using the PiPS trial data. Our preliminary analyses show the NNRD database could potentially serve as a reliable source of data on treatment costs and effects for future trial-based economic evaluations of neonatal interventions. Application to other trial-based economic evaluations where the NNRD has been used as a source of data would allow the potential of this resource to be explored for trial-based economic evaluations.

The methodology outlined in this paper is based on the incremental monetary net benefit statistic as the final economic endpoint of interest in the economic evaluations. This enabled the joint endpoints of clinical outcome and cost to be transformed to a univariate scale whilst accounting for the correlations between patient-level costs and effects between datasets. The transformation also allows for assessment of agreement to be conducted when costs and outcomes are measured on different scales (for example where cost is a continuous variable and the clinical outcome is binary as is the case in our illustrative example). Rather than transforming costs and health outcomes to the same scale, an alternative and potentially more attractive strategy would be to assess the agreement between observed resource use and clinical outcome variables when multiple sources of healthcare utilization and clinical outcome data are available. This is similar to assessment of agreement between measurements of a multivariate response such as blood pressure measurements with two pressure readings (diastolic and diastolic), and repeated measurements where outcomes are measured over time. Methods have been proposed in the literature extending the approach by Lin [18] to assessment of agreement of more complex data structures such as repeated measurement problems and multivariate response variables measured on the continues scale [5, 14, 16, 17]. These methods can, in principle, be adapted for assessment of agreement between two sources of data on treatment costs and effects. We have not however done so in our study because whilst healthcare costs are measured on the continuous scale, the clinical endpoint of interest in the PiPS trial example that serves to motivate our approach is a binary outcome (i.e., whether or not an infant avoids an episode of sepsis). It is not immediately obvious how to adapt these multivariate techniques for assessing agreement between outcomes measured on different scales. Further methodological work exploring the feasibility of assessing agreement involving multivariate mixed outcomes where the outcomes measured are of different data types and measured on different scales would present a useful advancement of the methodology presented here.

Our methodological framework assumes that the cost-effectiveness threshold is not kinked despite evidence from O’Brien et al. [25] that a kinked threshold better reflects asymmetrical individual preferences found in empirical studies of consumer’s willingness to pay for health changes, which would in turn justify different decision rules in the north-east and south-west quadrants of the cost-effectiveness plane [10]. Further research is required to assess how the methodological framework presented here might be extended in the presence of a kinked cost-effectiveness threshold.

Finally, how might the approach outlined above be used in practice? Our goal in this paper is to develop a methodology for assessing the level of agreement between the final economic endpoints of interest in trial-based economic evaluations. The method should not be applied directly to economic evaluations based on observational data or alongside other non-randomized study designs as the results of such analyses could be biased by the lack of randomization. This can propagate into biased estimates of agreement. Further work is required to develop methods that allow the level of agreement between cost-effectiveness outcomes to be assessed whilst appropriately accounting for potential imbalances in the distribution of confounding factors between the treatments being compared in the economic evaluation.

## Appendix A: variance of the incremental net (monetary) benefit

To derive the variance of the difference between two incremental net monetary benefits, we first derive the variance of the net benefit in terms of variances and covariances between costs and effects within trial arms. Let $C_{\text{A}}$, $E_{\text{A}}$, $\sigma_{{C_{\text{A}} }}$,$\sigma_{{E_{\text{A}} }}$ and $\rho_{{C_{\text{A}} E_{\text{A}} }}$ represent population level estimates of the mean costs, mean effects, standard deviation of costs, standard deviation of effects and covariance between costs and effects in intervention arm *A* (taken as control). We have $C_{\text{B}}$, $E_{\text{B}}$, $\sigma_{{C_{\text{B}} }}$,$\sigma_{{E_{\text{B}} }}$ and $\rho_{{C_{\text{B}} E_{\text{B}} }}$ as the corresponding quantities in intervention arm *B*, respectively. By definition of the incremental net benefit at a specified cost-effectiveness threshold, $\lambda$, we have

6 $$\beta_{\lambda } = \lambda \Delta E - \Delta C = \lambda (E_{\text{B}} - E_{\text{A}} ) - (C_{\text{B}} - C_{\text{A}} ).$$

Taken variances of both sides of Eq. (6), we have

7 $$\begin{aligned} \text{var} (\beta_{\lambda } ) & = \lambda^{2} \text{var} (\Delta E) + \text{var} (\Delta C) - 2\lambda \text{cov} (\Delta E,\Delta C) \\ & = \lambda^{2} \text{var} (E_{\text{B}} - E_{\text{A}} ) + \text{var} (C_{\text{B}} - C_{\text{A}} ) - 2\lambda [(E_{\text{B}} - E_{\text{A}} ),(C_{\text{B}} - C_{\text{A}} )]. \\ \end{aligned}$$

The variance terms on the right hand side of Eq. (7) are given by $var(E_{\text{B}} - E_{\text{A}} ) = \sigma_{{E_{\text{B}} }}^{2} + \sigma_{{E_{\text{A}} }}^{2}$ and $var(C_{\text{B}} - C_{\text{A}} ) = \sigma_{{C_{\text{B}} }}^{2} + \sigma_{{C_{\text{A}} }}^{2}$, and the covariance term by

8 $$\begin{aligned} {\text{cov[}}(E_{\text{B}} - E_{\text{A}} ),(C_{\text{B}} - C_{\text{A}} ) ]& = {\text{cov(}}E_{\text{B}} ,C_{\text{B}} )- {\text{cov(}}E_{\text{B}} ,C_{\text{A}} ) \\ & \quad- {\text{cov(}}E_{\text{A}} ,C_{\text{B}} )+ {\text{cov(}}E_{\text{A}} ,C_{\text{A}} )\\ & = {\text{cov(}}E_{\text{B}} ,C_{\text{B}} )+ {\text{cov(}}E_{\text{A}} ,C_{\text{A}} ) \\ & = \rho_{{E_{\text{A}} C_{\text{A}} }} + \rho_{{E_{\text{B}} C_{\text{B}} }} . \\ \end{aligned}$$

The two middle terms on the right hand side of the first line of Eq. (8) are zero because treatment arms in trials with a parallel-group design (i.e., no treatment switching or cross-over effects) are independent. Substituting the expressions for the variance and covariance terms derived in Eq. (8) into Eq. (7) gives the variance of the incremental net benefit in terms of the arm-specific variances and covariances between costs and effects:

9 $$var(\beta_{\lambda } ) = \lambda^{2} (\sigma_{{E_{\text{B}} }}^{2} + \sigma_{{E_{\text{A}} }}^{2} ) + (\sigma_{{C_{\text{B}} }}^{2} + \sigma_{{C_{\text{A}} }}^{2} ) - 2\lambda (\rho_{{E_{\text{A}} C_{\text{A}} }} + \rho_{{E_{\text{B}} C_{\text{B}} }} ).$$

## Appendix B: Covariance between two incremental net (monetary) benefits

The variance of the difference between two incremental net benefits is derived by taking variances of both sides of the expression $\omega_{\lambda } = \beta_{2\lambda } - \beta_{1\lambda }$ given by Eq. (1):

10 $$var(\omega_{\lambda } ) = \text{var} (\beta_{1\lambda } ) + \text{var} (\beta_{2\lambda } ) - 2\text{cov} (\beta_{1\lambda } ,\beta_{2\lambda } ).$$

The variance terms on the right-hand side of Eq. (10) are given by Eq. (9) for the *i*th dataset (*i* = 1, 2), so all we need is an expression for the covariance term $\text{cov} (\beta_{1\lambda } ,\beta_{2\lambda } )$. Now from the definition of the incremental net benefit (6), we have

11 $$\begin{aligned} {\text{cov(}}\beta_{1\lambda } ,\beta_{2\lambda } )& = \text{cov} [(\lambda \Delta E_{1} - \Delta C_{1} ),(\lambda \Delta E_{2} - \Delta C_{2} )] \\ & = {\text{cov(}}\lambda \Delta E_{1} ,\lambda \Delta E_{2} )- {\text{cov(}}\lambda \Delta E_{1} ,\Delta C_{2} ) \\ & \quad- {\text{cov(}}\lambda \Delta E_{2} ,\Delta C_{1} )+ {\text{cov(}}\Delta C_{1} ,\Delta C_{2} ).\\ \end{aligned}$$

The first term on the right-hand side of Eq. (11) is

12 $$\begin{aligned} {\text{cov}}\left( {\lambda \Delta E_{1} ,\lambda \Delta E_{2} } \right) & = {\text{cov}}\left[ {\lambda (E_{1B} - E_{1A} ),\lambda (E_{2B} - E_{2A} )} \right] \\ & = \lambda^{2} ({\text{cov(}}E_{1B} ,E_{2B} )- {\text{cov(}}E_{1B} ,E_{2A} ) & \quad- {\text{cov(}}E_{1A} ,E_{2B} )+ {\text{cov(}}E_{1A} ,E_{2A} )) \\ & = \lambda^{2} ({\text{cov(}}E_{1B} ,E_{2B} )+ {\text{cov(}}E_{1A} ,E_{2A} )) \\ & = \lambda^{2} (\rho_{{E_{1A} E_{2A} }} + \rho_{{E_{1B} E_{2B} }} ). \\ \end{aligned}$$

The remaining terms on the right-hand side of Eq. (11) can be derived in a similar manner:

13 $$\begin{aligned} {\text{cov(}}\lambda \Delta E_{1} ,\Delta C_{2} ) & = {\text{cov}}\left[ {\lambda (E_{1B} - E_{1A} ),(C_{2B} - C_{2A} )} \right] \\ & = {\text{cov(}}\lambda E_{1B} ,C_{2B} )- {\text{cov(}}\lambda E_{1B} ,C_{2A} ) \\ &\quad- {\text{cov(}}\lambda E_{1A} ,C_{2B} )+ {\text{cov(}}\lambda E_{1A} ,C_{2A} )\\ & = {\text{cov(}}\lambda E_{1B} ,C_{2B} )+ {\text{cov(}}\lambda E_{1A} ,C_{2A} )\\ & = \lambda (\rho_{{E_{1A} C_{2A} }} + \rho_{{E_{1B} C_{2B} }} ). \\ \end{aligned}$$

14 $${\text{cov}}(\lambda \Delta E_{2} ,\Delta C_{1} ) = \lambda (\rho_{{E_{2A} C_{1A} }} + \rho_{{E_{2B} C_{1B} }} ),$$

15 $${\text{cov(}}\Delta C_{2} ,\Delta C_{1} )= \rho_{{C_{2A} C_{1A} }} + \rho_{{C_{2B} C_{1B} }} .$$

Substituting the results of Eqs. (12)–(15) into Eq. (11) gives Eq. (16) as the covariance between two incremental net (monetary) benefits evaluated at a cost-effectiveness threshold, $\lambda$:

16 $${\text{cov(}}\beta_{1\lambda } ,\beta_{2\lambda } )= \lambda^{2} (\rho_{{E_{1A} E_{2A} }} + \rho_{{E_{1B} E_{2B} }} ) - \lambda (\rho_{{E_{1A} C_{2A} }} + \rho_{{E_{1B} C_{2B} }} ) - \lambda (\rho_{{E_{2A} C_{1A} }} + \rho_{{E_{2B} C_{1B} }} ) + (\rho_{{C_{2A} C_{1A} }} + \rho_{{C_{2B} C_{1B} }} ).$$

When carrying out the analysis in practice, all parameters in Eqs. (6)–(16) are replaced with their sample counterparts $\hat{C}_{A}$, $\hat{E}_{A}$, $\hat{\sigma }_{{C_{A} }}$,$\hat{\sigma }_{{E_{A} }}$ and $\hat{\rho }_{{C_{A} ,E_{A} }}$ in arm *A* and $\hat{C}_{B}$, $\hat{E}_{A}$, $\hat{\sigma }_{{C_{B} }}$,$\hat{\sigma }_{{E_{B} }}$ and $\hat{\rho }_{{C_{B} ,E_{B} }}$ in arm *B*. The square of the standard error of the incremental net benefits in equation (2) are obtained by dividing the arm-specific variance and covariance terms on the right hand side of Eqs. (6)–(16) by *N*_*A*_ and *N*_*B*_, the number of individuals in treatment arms *A* and *B* respectively.

## Appendix C: methods of the PiPS trial economic evaluation

### Study population

#### Probiotics in Preterm Infants Study (PIPS) trial

Probiotics in Preterm Infants Study (PIPS) was a multi-center blinded randomized placebo-controlled trial designed to test the effectiveness of the probiotic* Bifidobacterium breve* BBG-001 to reduce NEC, late-onset sepsis, and death in preterm infants. Infants born between 23 weeks and 0 days and 30 weeks and 6 days of gestation with written parental consent were eligible for recruitment.

#### The National Neonatal Research Database (NNRD)

The National Neonatal Research Database (NNRD) has been created through the collaborative efforts of neonatal services across the country to be a national resource. The NNRD contains a defined set of data items (the Neonatal Dataset) that have been extracted from the Badger.net neonatal electronic health record of all admissions to NHS neonatal units. Badger.net is managed by Clevermed Ltd, an authorized NHS hosting company. The Neonatal Dataset is an approved NHS Information Standard (ISB1575). Contributing neonatal units are known as the UK Neonatal Collaborative. Variables that allowed for the creation of comparable resource use Items (directly available or derivable) were extracted from the NNRD

#### Combined dataset

An additional dataset was created by selecting a variable from either PIPS or NNRD that represented a resource use item more accurately.

For the purposes of our study, only those infants were analyzed for whom there was data available from both the PIPS trial and the NNRD.

### Type of economic evaluation, study perspective, and time horizon

The economic evaluation took the form of a cost-effectiveness analysis in which we estimated the incremental costs (Δ*C*) and incremental effects (Δ*E*) attributable to probiotic (B breve BBG) in preterm infants, with reference to a placebo, and expressed each in terms of an incremental cost-effectiveness ratio (ICER; Δ*C*/Δ*E*). Estimates of cost-effectiveness were made for the three primary clinical outcomes (any episode of NEC, any case of Sepsis, death before discharge from hospital), and for one secondary outcome, which was a composite of the three primary outcomes. The economic evaluation was conducted from a health system perspective and consequently only direct costs to the NHS were included [24]. The time horizon of the study was birth to discharge or death, whichever was earlier.

### Measurement of resource use and costs

Relevant resource items were integrated into the trial data collection instruments described previously. The neonatal and maternal data collection forms captured a comprehensive profile of resource use by each infant, encompassing length of stay by intensity of care, surgeries, investigations, procedures, transfers, and post-mortem examinations until final hospital discharge or death (whichever was earliest). Variables that allowed for comparison of selected resource use items in the PIPS data directly or through derivation were extracted from the NNRD. Resource inputs were valued based on data collated from secondary national tariff sets [8, 9]. All costs were expressed in pounds sterling and reflected values for the financial year 2012–13.

The total length of stay (total inpatient hospital days) was computed as the total number of hospital days until first discharge to home or death. Postnatal costs for the mothers were based on the method of delivery available in the data source and costs assigned using data from the NHS Reference Costs trusts schedule 2012/13 [9]. Information was available on time spent in the neonatal unit by level of care (normal, transitional, special, high dependency or intensive), by varying level of detail from both data sources. The cost of neonatal care was calculated for each infant by multiplying the length of stay in normal care (where available), transitional care (where available), special care, high dependency care, or intensive care by the per diem cost of the respective level of care using data from the NHS Reference Costs trusts schedule 2012/13 [9]. The costs of surgeries and procedures were calculated by assignment of surgical procedures to relevant Healthcare Resource Group (HRG) codes and application of unit costs from national tariffs [9]. Transfers were recorded whenever an infant was transported between specialist hospitals for neonatal critical care, and were valued using costs from the NHS Reference Costs trusts schedule 2010/11 [9]. Post-mortem costs were based on data from secondary sources [30]. Non-routine investigations excluded from these per diem costs were valued using a combination of primary and secondary costs. Where these costs were not available from national tariffs, clinicians were asked to identify the staff and material inputs required for these investigations. Staff time was valued using the Unit Costs of Health and Social Care tariffs [8].

### Cost-effectiveness analytical methods

Neonatal characteristics and resource use items were summarized by trial arm (placebo or *B. breve BBG*). Differences between groups were analyzed using *t* tests for continuous variables and *χ* ^2^ test for categorical variables. Mean (standard error (SE) costs by cost category and mean (SE) total costs were estimated by trial arm and comparisons were carried out using Student’s *t* tests.

Cost effectiveness was expressed as incremental cost per (1) adverse perinatal outcome avoided. Nonparametric bootstrapping, involving 1000 bias-corrected replications of each of the incremental cost effectiveness ratios, was used to calculate uncertainty around all cost-effectiveness estimates [35]. This was represented on four quadrant cost-effectiveness planes. Decision uncertainty was addressed by estimating net benefit statistics and constructing cost-effectiveness acceptability curves across cost-effectiveness threshold values of between £0 and £70,000 for the health outcomes of interest. A series of sub-group analyses repeated all analyses by selected sub-groups for the primary and secondary cost-effectiveness outcomes. All analyses were estimated using Stata version 12 [32] and R version 2.01 [28].
